# Valve-Like Outflow System Behavior With Motion Slowing in Glaucoma Eyes: Findings Using a Minimally Invasive Glaucoma Surgery–MIGS-Like Platform and Optical Coherence Tomography Imaging

**DOI:** 10.3389/fmed.2022.815866

**Published:** 2022-04-29

**Authors:** Murray Johnstone, Chen Xin, Ted Acott, Janice Vranka, Joanne Wen, Elizabeth Martin, Ruikang K. Wang

**Affiliations:** ^1^Department of Ophthalmology, University of Washington, Seattle, WA, United States; ^2^Department of Ophthalmology, Tongren Hospital, Beijing, China; ^3^Department of Ophthalmology, Casey Eye Institute, Portland, OR, United States; ^4^Department of Ophthalmology, Duke Eye Center, Durham, NC, United States; ^5^Department of Ophthalmology, Indiana University, Indianapolis, IN, United States; ^6^Department of Bioengineering, University of Washington, Seattle, WA, United States

**Keywords:** intraocular pressure regulation, aqueous outflow regulation, trabecular meshwork, MIGS, collector channels, aqueous valves, glaucoma, OCT

## Abstract

**Purpose:**

This study aimed to investigate anatomic relationships and biomechanics of pressure-dependent trabecular meshwork and distal valve-like structure deformation in normal and glaucoma eyes using high-resolution optical coherence tomography (HR-OCT).

**Methods:**

We controlled Schlemm’s canal (SC) pressure during imaging with HR-OCT in segments of three normal (NL) and five glaucomatous (GL) *ex vivo* eyes. The dissected limbal wedges were studied from 15 locations (5 NL and 10 GL). A minimally invasive glaucoma surgery (MIGS)-like cannula was inserted into the SC lumen, whereas the other end was attached to a switch between two reservoirs, one at 0, the other at 30 mm Hg. A steady-state pressure of 30 mm Hg was maintained to dilate SC and collector channels (CC) during 3D volume imaging. The resulting 3D lumen surface relationships were correlated with internal structural features using an image mask that excluded tissues surrounding SC and CC. While imaging with HR-OCT, real-time motion responses in SC and CC areas were captured by switching pressure from 0 to 30 or 30 to 0 mm Hg. NL vs. GL motion differences were compared.

**Results:**

Lumen surface and internal relationships were successfully imaged. We identified SC inlet and outlet valve-like structures. In NL and GL, the mean SC areas measured at the steady-state of 0 and 30 mm Hg were each significantly different (*p* < 0.0001). Synchronous changes in SC and CC lumen areas occurred in <200 ms. Measured SC area differences at the steady-state 0 and 30 mmHg, respectively, were larger in NL than GL eyes (*p* < 0.0001). The SC motion curves rose significantly more slowly in GL than NL (*p* < 0.001). Pressure waves traveled from the cannula end along the SC lumen to CC and deep intrascleral channels.

**Conclusion:**

HR-OCT provided simultaneous measurements of outflow pathway lumen surfaces, internal structures, and biomechanics of real-time pressure-dependent dimension changes. We identified SC inlet and outlet valve-like structures. GL tissues underwent less motion and responded more slowly than NL, consistent with increased tissue stiffness. A MIGS-like shunt to SC permitted pulse waves to travel distally along SC lumen and into CC.

## Introduction

Glaucoma is a leading cause of blindness with control of intraocular pressure (IOP), the only treatable risk factor (1). Aqueous outflow regulation determines IOP but becomes abnormal in glaucoma. Schlemm’s canal (SC) directed minimally invasive glaucoma surgeries (MIGS) rely on the hypothesis that the trabecular meshwork (TM) controls resistance to aqueous flow (2). If the hypothesis explained flow control, bypassing the TM with device placement, disruption, or ablation would reduce IOP to near episcleral venous pressure levels. However, these MIGS do not consistently achieve low pressures (3), suggesting that pathways distal to the TM also play an important role (4).

Traditionally, challenges in imaging have limited the study of the biomechanics of the outflow pathways distal to the TM. However, recent technical advances have made such imaging studies more feasible. The advances in relevant distal pathway imaging include viscoelastic injection with scanning electron microscopy (5–7), ribbon-scanning technologies (8), 3D Micro CT (9), and 2-photon fluorescence (10, 11). *In vivo*, human advances include the use of spectral-domain optical coherence tomography (SD-OCT) to demonstrate pressure-dependent SC lumen changes (12, 13) and phase-sensitive OCT (PhS-OCT) (14–16) to detect TM motion that determines the changes in SC dimension.

The TM is poised between the AC and SC in a prestressed, tensionally integrated configuration at a physiologic IOP (7). The equipoise results because IOP acts as a force causing TM distention into SC, while ciliary muscle tension combined with TM lamellae elastance acts as a counterforce. Pressure-dependent TM distension and recoil depend on the TM elastance properties to store and release elastic energy (7).

Reversal of pressure gradients *in vivo* using pressure on the episcleral veins or postural inversion causes SC to fill with blood (17–19). Histologic studies confirm that SC widely dilates, fills with blood, and the TM collapses (20). Inducing pressure gradients from the AC requires OCT measurements through the thickness of the sclera and results in too low a resolution to assess details of TM movement. Our system uses OCT to image from the TM surface and provides sufficient resolution to measure TM motion (21). Transtrabecular pressure gradients with OCT imaging can only be generated from within SC under these circumstances, a less familiar but physiologic phenomenon (22).

There are three questions arising related to pressure-dependent TM motion. The first question is how the TM responds to mean changes in IOP that maintain the TM positional equipoise between the AC and SC *in vivo*. Studies using *in vivo* fixation in living primates with normal EVP and ciliary body tension provide guidance (20, 23–27). The second question is how the TM responds to oscillatory waves resulting from pulse-induced choroidal volume changes, which we study in patients with phase-OCT (PhS-OCT) (14, 15, 28) and experimentally in the laboratory with high-resolution OCT (21). The third question is how the TM responds to transients that result from blinking or eye movements, which we explore in an *ex vivo* environment using experimentally controlled pressures and 2D real-time TM motion measurements with high-resolution OCT (29).

The human eye continuously experiences pulse-dependent oscillations of about three mm Hg. Blinking and eye movements result in multiple 10 mm Hg transient IOP spikes above the mean baseline IOP each minute. Phs-OCT (30, 31) advances now permit imaging of rapid cardiac cycle-dependent TM motion *in vivo* in humans (14, 15), finding a significant reduction of TM motion in patients with glaucoma (16).

Loss of TM motion in glaucoma is consistent with this report’s key concepts: the aqueous outflow system controls IOP by acting as a regulatory pump-conduit system (6, 7, 32, 33). IOP-dependent tissue motion provides sensory input, while alterations in pathway dimensions serve the motor function of regulating flow. In glaucoma, the system fails due to increased TM stiffness that limits normal motion.

The aorta experiences constant oscillatory stresses like the TM. The walls are composed of elastin and collagen; the elastin component is ∼100 times more distensible. With increasing age, there is elastin fragmentation, replacement with collagen, loss of ability to recoil, and an associated increase in distension of the vessel wall (34). The dimension changes are not trivial—the elastin fragmentation-collagen replacement results in an aorta diameter increase of 50% between age 40 and 70. The TM is a similar distending vessel wall that both stiffens and distends. TM progressive distention into SC leads to apposition and eventual adhesion between SC walls in glaucoma (35, 36).

Our findings are consistent with clinical studies that point to outflow tissue stiffening with reduced motion and slowed aqueous flow. Aqueous veins exhibit pulsatile flow, but the flow slows and eventually stops in glaucoma (37, 38). Similarly, the aqueous influx test that results in an enhanced pulsatile flow response in normal subjects is absent in glaucoma (37, 39–41). Moreover, clinical gonioscopy studies observe the presence and rapidity of blood reflux into SC requiring TM motion. The studies separate glaucoma from normal patients based on the loss of TM motion (17, 19, 42). In addition, PhS-OCT imaging demonstrates reduced TM motion in glaucoma patients (16). The studies point to increased TM stiffness, reduced TM motion, and progressive clinically observable pulsatile flow abnormalities in glaucoma.

Laboratory studies with high-resolution OCT (HR-OCT) provide examples of the TM and the walls of the collector channels (CC) undergoing substantial, synchronous shape changes in response to changing SC pressure gradients. The shape changes occur in non-human primates (21) and human eyes (43–46) but warrant more in-depth study. SC pressure on the TM walls represents a loading force that induces stresses resulting in strain altering the bulk responses of the TM lamellae. While HR-OCT has sufficient resolution to assess the changes in SC and CC area reflective of bulk TM and CC entrance tissue motion, it is not adequate to detect the motion of the juxtacanalicular region or SC epithelium (SCE) at the cellular level.

Studies using atomic force microscopy (AMF) implicate changes in outflow system stiffness in the glaucoma process (47, 48). Measurements involve studying properties within ∼1 μm of SC inner wall endothelium (48). One study compared stiffness in the same human tissues derived from the measurements by AMF or HR-OCT via finite element analysis (FEM). The elastic modulus of normal TM estimated by OCT measurement was 70 ± 20 kPa (mean ± *SD*) vs. glaucomatous tissue (98 ± 19 kPa). TM stiffnesses measured by AFM in normal was (1.37 ± 0.56 kPa) vs. glaucoma (2.75 ± 1.19 kPa). Glaucoma vs. normal differences were not statistically significant by either technique. However, the kPa of the AMF and HR-OCT/FEM approach differed markedly (45). AFM measures localized regional stiffness of SC inner wall region vs. OCT that assesses bulk motion of the entire TM under conditions of a pressure-induced load, which may explain the differences.

Our HR-OCT platform permits studying steady-state 3-dimensional (3D) SC-CC relationships, internal architecture, and motion in the same outflow tissues. The technology also enables comparisons of global motion differences between normal and glaucomatous outflow tissues.

Here, we explored the hypothesis that both the TM and distal outflow system function in unison to provide an IOP regulatory mechanism in human eyes, a function that becomes abnormal in glaucoma because of alterations in tissue elastance/stiffness. We used an experimental MIGS-type platform to test the hypothesis. The platform bypasses the TM, permitting direct communication of pressure gradients to SC. Regulation of SC pressure as the experimental variable allows systematic control of TM pressure gradients during real-time imaging.

Goals to determine the success of the experimental platform include the ability (1) to compare the 3D surface and internal architecture of the outflow channels under different steady-state pressure conditions, (2) to determine whether SC and CC real-time pulse-dependent changes in lumen dimension can be quantified, and are synchronous, and (3) to determine whether there are differences in motion responses between normal and glaucomatous eyes.

## Materials and Methods

**FIGURE 1:**
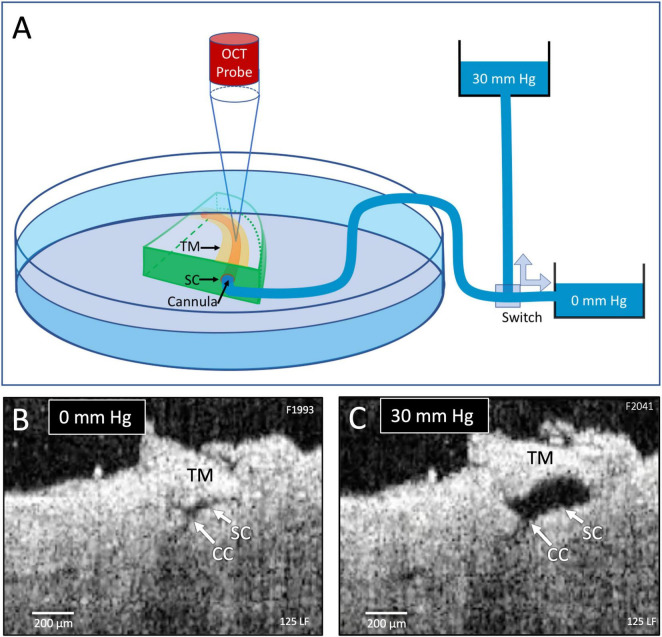
Experimental setup and real-time pressure-dependent optical coherence tomography (OCT) images. **(A)** A tissue wedge in a Petri dish with the trabecular meshwork (TM) facing upward during high-resolution OCT imaging. A micromanipulator guides one end of a cannula to the wedge and holds it securely in Schlemm’s canal (SC). The opposite end of the cannula leads to a valve that switches between two reservoirs, one at 0 mm Hg and the other at 30 mm Hg. OCT imaging is initiated before switching reservoirs and continues until achieving a new steady-state tissue configuration. **(B,C)** OCT images at 0 and 30 mm Hg, respectively, from the distal SC location, are noted in Figure 2. Both SC and collector channels (CC) collapse at 0 mm Hg but markedly enlarge when pressure increases in SC.

Human eye bank eyes (3 NL and 5 GL, (Mean age 80.3 ± 9.4 years), (Sex 5F/3M), (White 4, Other 4) were used in the study and prepared as described in detail in Supplementary Data. In brief, wedges of limbal tissue, each incorporating 1/8 of the limbal circumference, were prepared and immersed in Hank’s Balanced Salt Solution in a Petri dish (21) (Figure 1). To assess whether differences in the rapidity of motion were present in glaucoma eyes, we imaged locations in a subset of five wedges in the NL and ten in the GL eyes. A specially constructed steeply tapered MIGS-sized cannula (21) formed from flexible PE 60 tubing with an outside diameter of 1.22 mm, a taper length of 4.5 mm, and a final diameter of 130–150 μm fit tightly into the SC entrance.

**FIGURE 2:**
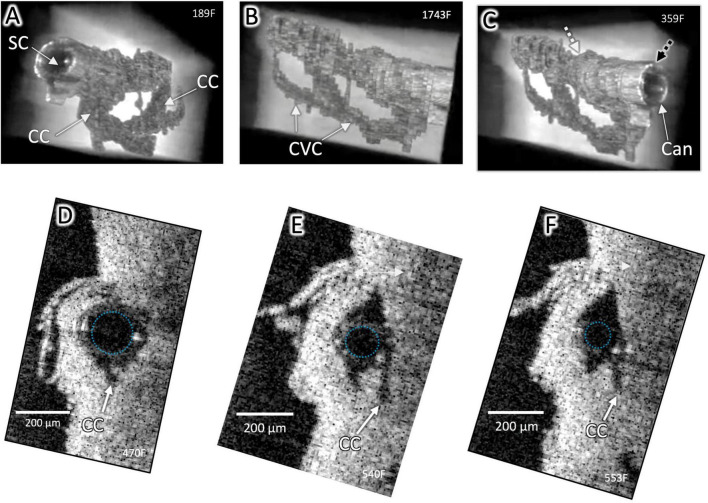
Surface architecture and internal structure with a cannula in Schlemm’s canal. High-resolution OCT imaging permits delineation of the lumen boundaries of the outflow system after segmentation to eliminate surrounding structures in individual OCT B-scans and reconstruction with Amira 3D volumetric software. A cannula (Can) is visible in SC in **(A–C)**, and the cannula’s internal diameter is outlined in **(D–F)**. CC connect with an intrascleral circumferential-oriented vascular compartment (CVC) adjacent to and parallel with SC. Cannula location in **(C)** (Black dashed arrow) shown in cross-section in **(D)**. Location of end of cannula denoted by the white arrow in **(C)** and cross-section in **(F)**. The striped arrow distal to the cannula in **(C)** is the location of Figure 1 **(B,C)**. The outside diameter (OD) of the cannula at (C) was 241 μm and at **(D)** was 178 μm, typical of stent sizes and here occludes the CC entrance. Distally, the smaller cannula diameter no longer occludes CC. Both CC and CVC are distended distal and proximal to the cannula end, demonstrating a pathway for fluid entry to the distal system from the cannula end (Supplementary Video 1).

The cannula diameter at the SC entry site was approximately 200 μm (Figure 2). The other end of the canal led to a valve that switched between one reservoir providing 0 and another at 30 mm Hg hydrostatic pressure (Figure 1C). We established a steady-state pressure of 0 mm Hg while imaging with the HR-OCT system (Figure 1B). Then we switched the valve to redirect the pressure to the 30 mm Hg pressure reservoir while maintaining OCT imaging until a new steady-state was achieved (Figure 1C). Protocol reversal changed the hydrostatic pressure head from 30 to 0 mm Hg.

We positioned the OCT probe to face the TM surface to avoid scleral light scattering and vessel-related shadowing. The OCT system was a spectral-domain (SD-OCT) setup that employed a 1,310 nm wavelength broadband light source and a spectrometer detector running at 92 kHz, providing an imaging speed of 92,000 A-scans/s. Spatial resolution in each scan was 5.2 μm and 7.5 μm in the axial and lateral dimensions, respectively. At the measured light power on the sample of 5 mW, a system sensitivity of 105 dB was measured. We imaged locations from five NL and ten GL eyes.

We assessed steady-state relationships by 3D volumetric OCT scans of 3 × 3 mm (400 × 400 A-scan density) areas stitched together, providing a composite of the tissue appearance of entire limbal wedges. In the same specimens, a mask eliminated tissues surrounding the lumen of outflow system structures, permitting same-specimen characterization of surface architecture of luminal channels (see details in Supplementary Data).

Dynamic 2 dimensional (2D) cross-sectional OCT images provided tissue motion information. The system was operated at repeated scanning mode in the experiments, i.e., continuously capturing OCT B-scan images over time. The A-scan density was 400 in each B-scan. The imaging speed was 200 B-scans per second, providing a time interval between adjacent B-scans of 5 ms. We used time-lapse sequences of OCT cross-sectional images to capture regions subject to pressure change.

An algorithm was used to binarize the SC and CC lumen vs. surrounding tissue from the OCT images to acquire the motion information. There were five measurements that were then made at each time point and averaged to improve the accuracy of quantitative SC and CC lumen areas. Data were normalized using maximum SC and CC lumen areas as the reference. The resulting curves permitted assessment of the synchronicity of SC and CC lumen area changes.

Normalization permitted comparison of Schlemm’s canal area (SCA) data from 0–30 to 30–0 mm Hg measurements from 5 locations in the NL and 10 locations in the GL eyes. Averaging of data from each 5 ms time point permitted the development of curves of time-dependent SCA changes (analysis details are in Supplementary Data.) Paired *t*-tests assessed steady-state SC area dimension changes. Unpaired *t*-tests assessed differences in SC and CC area changes in NL vs. GL segments. All *t*-tests were 2-tailed. MANOVA evaluated the complex NL vs. GL SCA curves generated by the data. The statistical software package was JMP 13, version 13.2.

## Results

### Schlemm’s Canal Collector Channel and Minimally Invasive Glaucoma Surgery Relationships

**FIGURE 3:**
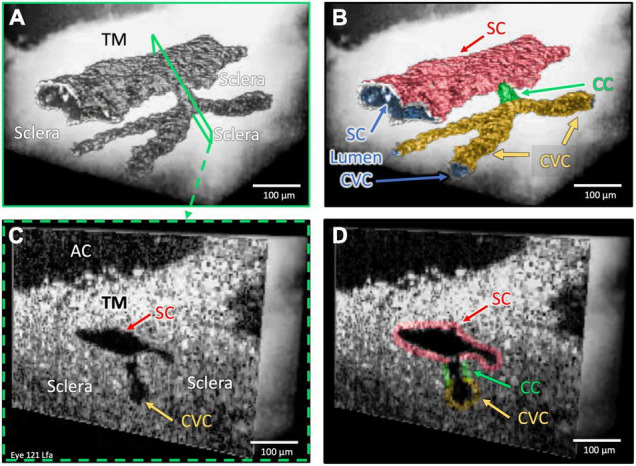
One collector channel: Architecture and internal relationships. Images **(A,B)** demonstrate the architecture of outflow structures obtained after segmentation of the lumens (dark appearance) visible in the OCT images. Cross-sections in **(C,D)** explore internal structure at a CC exiting SC that communicates with a CVC at the location of the blue slice in **(A)** and green outline in the color map in **(B)**. The lumen of the CC is a short segment oriented perpendicular to both SC and the CVC lumen. The SC and CVC lumens are oriented parallel to each other. SC, CC, and CVC lumen experience synchronous pressure-dependent compression and distention (Supplementary Video 2).

**FIGURE 4:**
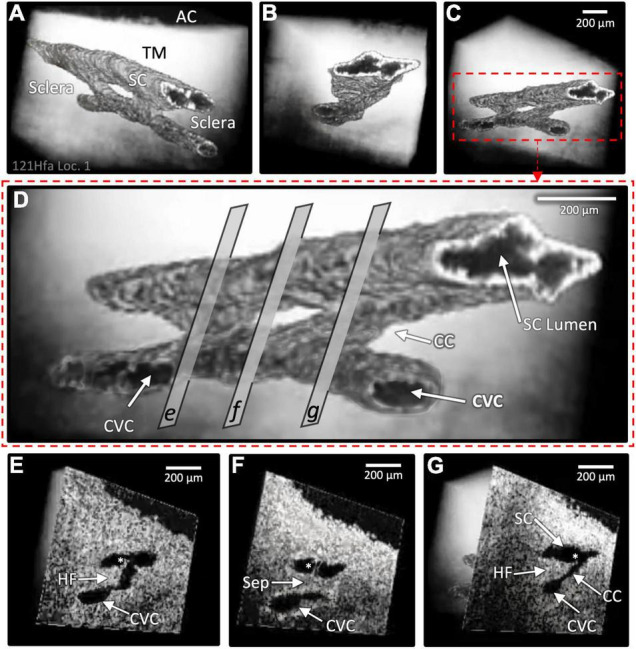
Two collector channels (CC): Architecture and internal relationships. Images **(A–D)** show the surface architecture of the outflow system in a region encompassing two CC that communicate with a circumferential vascular channel (CVC) parallel to SC, which were obtained after segmentation of lumens (dark appearance) in the 3D OCT images. Image **(D)** magnifies image **(C)** to show cross-sectional slices through regions **(E–G)** that represent locations of the 3D images in the lower panel of the cross-sectional images **(E–G)** at the exact location. Both CC lumens are short segments oriented perpendicular to the SC and CVC lumen; the lumens of SC and CVC are parallel. The region between the CC in **(F)** and the CVC is separated from SC by a septum (Sep) that appears as a void in **(D)**. The lumens of SC, the CC, and CVC experience synchronous motion in response to SC pressure changes. In **(E,G)**, asterisks identify hinged flaps (HF) at CC entrances. In **(F)**, a cylindrical attachment structure with lumen bridges between the TM and SC external wall (Supplementary Video 3).

**FIGURE 5:**
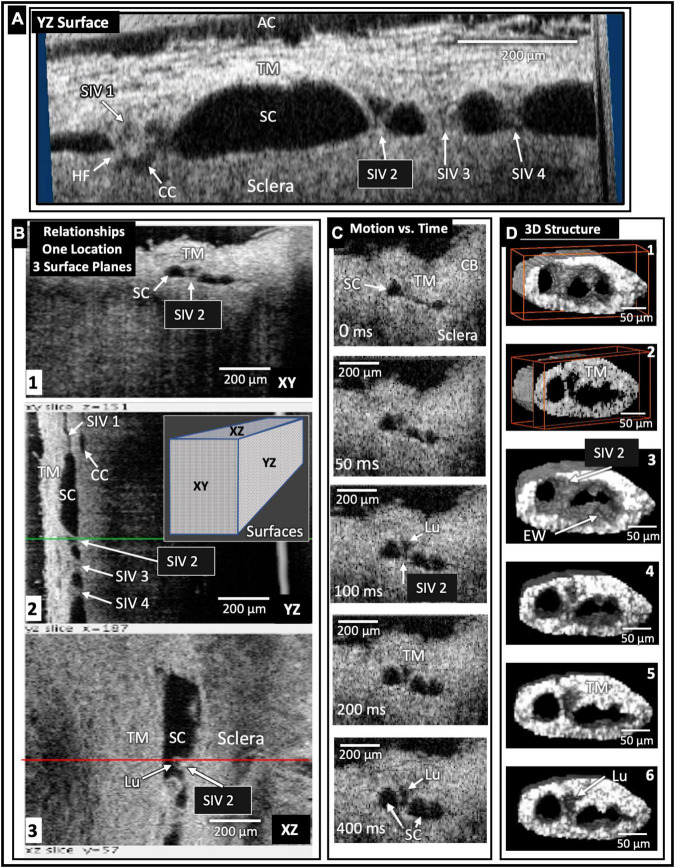
Schlemm’s canal inlet valve-like structure (SIV) 3D relationships and motion. Images from high-resolution OCT captured 3D volumes (XYZ) while maintaining 30 mm Hg SC hydrostatic pressure. Trabecular meshwork (TM), Schlemm’s canal (SC), the scleral or external wall (EW) of SC, and CC relationships, as well as SIV connections, are apparent in the XY, YZ, and XZ relationships of **(A,B)**. Four SIV crossing SC are visible in **(A)**. At SIV1, a hinged flap (HF) is visible at a CC entrance. In **(B)**, the SIV2 appearance in each plane at one location seen in XY, YZ (green line), and XZ (red line) provides an improved appreciation of relationships and lumen appearance compared with XY alone. In **(C)**, the motion was captured using high-resolution OCT 2D imaging while SC pressure changed from 0 to 30 mm Hg. Most of the SIV2 lumen volume change occurred in <200 ms. In **(D)**, a mask eliminated surrounding structures providing 3D images of the surface appearance **(D1)** and internal structure at increased depths as indicated by the red box advance into the volume **(D2)**. **(D3)** through **(D6)** show surface volumes at increasing depth. The funnel-shaped deformation of the TM and SIV lumen (Lu) exiting from the TM is consistent with the strain induced by pressure-dependent stresses on the connections between the TM, SIV, and SC external wall.

**FIGURE 6:**
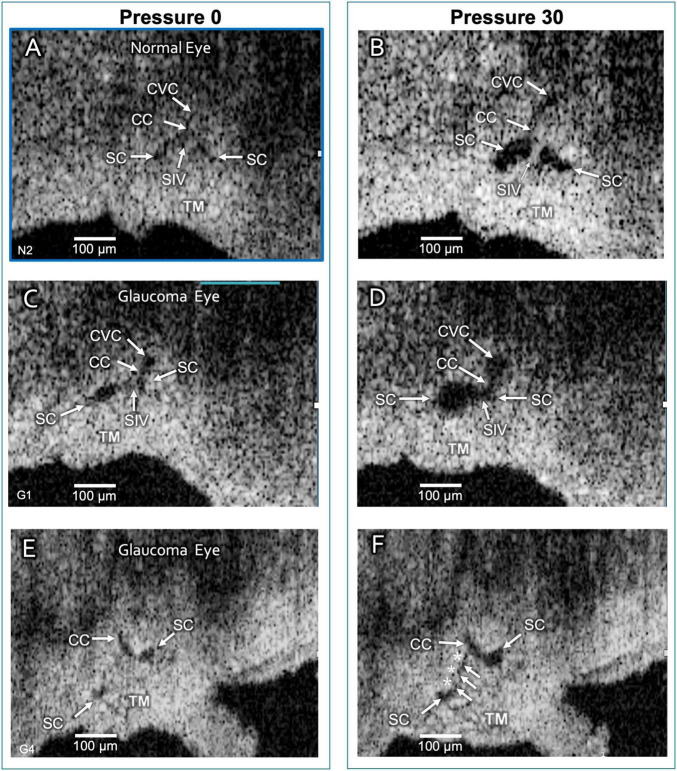
Schlemm’s canal and collector channel configuration changes. The 2D high-resolution cross-section (i.e., B-scan) OCT images in **(A,C,E)** are at 0 mm Hg hydrostatic reservoir pressure, while images in **(B,D,F)** are at 30 mm Hg. Schlemm’s canal (SC) spans between the paired arrows. In **(B,D)**, the lumen of SC, collector channel (CC) entrances, and the circumferentially-oriented vessel compartments (CVC) become markedly enlarged. Schlemm’s canal inlet valve-like structure (SIV) attachments between the trabecular meshwork (TM) and SC external wall elongation. **(E,F)** are from a glaucomatous eye. In **(F)**, the TM remains in the CC entrance. TM motion is marked, but adhesions between the TM (white arrows) and SC external wall (asterisks) prevent SC from opening.

The steeply tapered design of the cannula inserted into the SC entrance was crucial to permit SC occlusion while still maintaining flow. In the occlusion area at the entry site, the cannula diameter was approximately 200 μm, simulating MIGS SC device dimensions (Figures 2A–F). Examination of 3D images demonstrated regularly recurring CC arising from the external wall of SC (Figures 3–6). Images of the 3D architecture of SC revealed that the CC area lumen frequently joined a circumferentially-oriented vascular compartment (CVC) in the deep scleral plexus paralleling SC (Figures 3, 4, 6). Thin septa provided separation of the lumen of the CVC from the lumen of SC. Comparing the direct images and extracted lumen images highlights the septa role in creating a barrier between SC and the CVC. The septa appeared as void spaces between SC and the CVC in the lumen images (Figures 3, 4).

The cannula inserted into SC, simulating a MIGS stent, filled the SC lumen and occluded some CC entrances near the insertion site (Figures 2A,D). The SC lumen distal to the cannula (Figures 1B,C, 2C) was distended, as were CC and the more distal CVC. Even though a CC entrance adjacent to the MIGS cannula was occluded, distention was present in the underlying CC (Figures 2A,B). The pulse-dependent motion of the TM, CC entrance, and an intrascleral channel was visible ∼2 mm distal to the MIGS-like device despite an intervening SC constriction distal to the cannula (Figure 2 and Supplementary Video 1).

### Collector Channel Lumen Topographic Features and Internal Structure Relationships

The CC provided a short intrascleral conduit for aqueous passage through external wall septa before CVC entry (Figures 2–4). As a result, the junctions of the SC and CC lumen and those of the CC and CVC often had a nearly perpendicular relationship. A relatively rapid change in the direction of the pathways for flow resulted. Images of internal structures identified multiple hinged flaps at CC entrances (Figures 3C,D and Supplementary Videos 1–3). Septa edges where they join collector channel entrances appeared as hinged collagen flaps (Figures 4E–G). The mobile septa span between adjacent CCs (Figure 4). SC inlet valve-like structures (SIV) with a lumen arise from the SC inner wall endothelium of the TM and cross the canal, thereby forming attachments between the TM and the hinged collagen flaps at CC entrances (Figures 4F, 5A–D, 6B,D and Supplementary Videos 1–3).

### Motion Studies—Steady State Schlemm’s Canal Area Measurements

The initial and final steady-state SC, CC, and CVC lumen appearances differed markedly. Without pressure in SC (Figures 6A,C,E), their lumens varied from a potential space to a minimal size, but 30 mm Hg pressure resulted in lumen dilation (Figures 1B,C, 5, 6A–F). The *ex vivo* absence of ciliary muscle-induced stress and the associated small SC diameter area resulted in short SIV with an undetectable lumen at 0 mm Hg. As pressure increased, the SIV progressively elongated and developed a funnel-shaped lumen. The enlarging SIV lumen reached a new steady-state configuration within ≲200 ms (Figure 5).

The mean steady-state pressures of 0 and 30 mm Hg resulted in significantly different SC areas (SCA) in both NL (*p* < 0.0001) and GL (*p* < 0.0001). The 0 mm Hg SCA was 1,293 ± 120 μm^2^ in the NL, while in the GL tissue, it was 6,501 ± 428 μm^2^. However, the mean 30 mm Hg SCA values for NL and GL were similar at 13,843 μm^2^ and 15,024 ± 520 μm^2^, respectively. SCA underwent more extensive excursions in the NL than the GL locations (*p* < 0.0001) (see Supplementary Table 2 for steady-state details).

### Duration of Schlemm’s Canal and Collector Channels Area Changes Following 0–30 and 30–0 Pressure Changes

**FIGURE 7:**
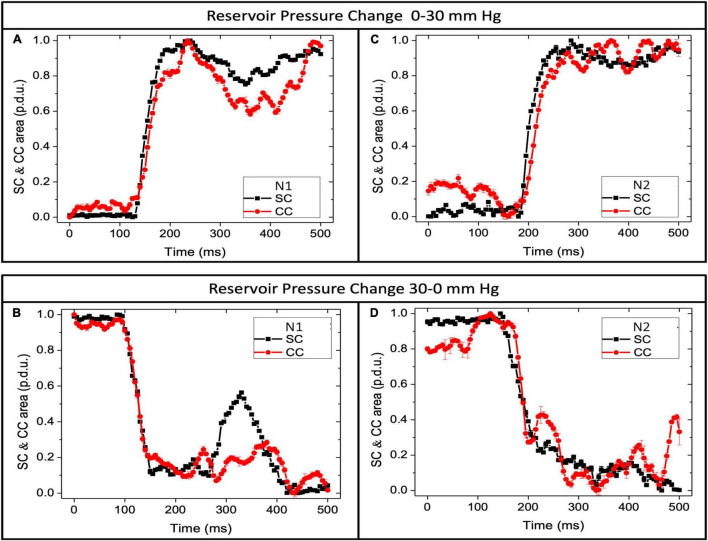
Schlemm’s canal and collector channels respond with pressure changes. Normal eye pressure responses. **(A,C)** SC area (SCA) and CC area (CCA) vs. time when reservoir pressure increases from 0 to 30 mm Hg. **(B,D)** SCA and CCA area when pressure decreases from 30 to 0 mm Hg. Y-axis is normalized data using the maximum area as the reference dimension. In both graphs, “p.d.u.” or procedure-defined dimensionless units define the area. The time to the first peak or trough was <200 ms. Oscillations after pressure changes were more pronounced when the pressure dropped from 30 to 0 mm Hg. SCA changes reflect trabecular meshwork mechanical properties, while CCA dimension changes reflect mechanical properties of collagenous structures at CC entrances. N is the subject’s eye. Supplementary Figure 2 documents synchronous motion of SCA and CCA at 3 locations in normal and 3 in glaucoma eyes.

A comparison of the duration of the TM and CC motion responses (Figure 7) demonstrated that at an SC pressure change (Δ) of 0–30 mm Hg, the mean time course of TM and CC motion to a new steady-state was 138.2 ± 8.4 and 137.8 ± 10.2 ms, respectively (*p* = 0.974). The Δ from 30 to 0 mm Hg was 178.3 ± 22.1, and 186.6 ± 20.7 ms, respectively (*p* = 0.789). The duration of motion of the ascending 0–30 and descending 30–0 mm Hg TM curves were not significantly different (*p* < 0.12), although the slower CC difference approached significance (*p* < 0.06) (see Supplementary Table 3 for confidence intervals and range).

### Dynamic Changes in Schlemm’s Canal Area Curves in Normal Compared With Glaucomatous Eyes

**FIGURE 8:**
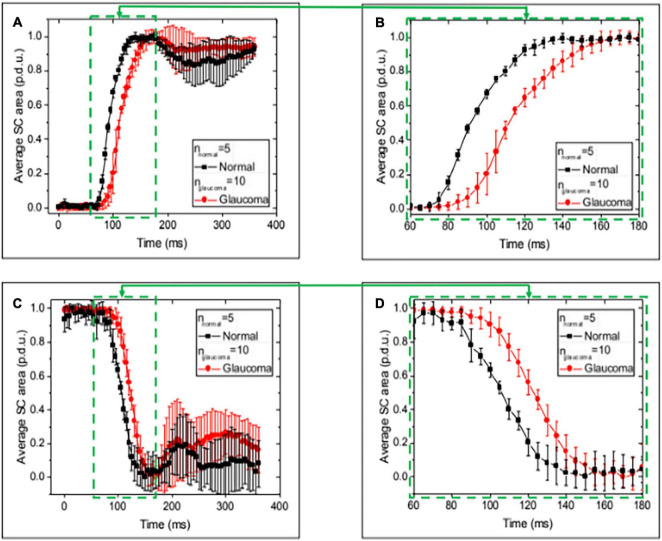
Normal and glaucoma eye responses to Schlemm’s canal pressure changes. Images **(A,C)** show normalized SC cross-sectional area vs. time over a 400 ms time interval while imaging at 5-s intervals during the 0–30 and 30–0 mm Hg changes in reservoir pressures. Data are the mean and SEM for normal and glaucoma eyes. **(B,D)** Are an enlarged view of areas **(A,C)**. The mean Schlemm’s canal area change occurred more rapidly in the normal than the glaucoma eyes at multiple time intervals. The total time to reach the peak or trough of motion was ≤200 ms. Oscillatory behavior remained a feature of the aggregated measurements at the end of both the rising and falling intervals. The mean time to a new peak or trough was ≤200 ms. Supplementary Table 2 provides a summary of baseline and final areas. MANOVA Δ for Normal vs. Glaucoma curves (*p* < 0.001).

When the pressure varied from 0–30 to 30–0 mm Hg, the aggregate dynamic motion curves of both NL and GL eye locations reached their initial endpoints within ≤ 200 ms (Figure 8), as did individual curves (Supplementary Figure 1). The aggregate SC area curves in GL eyes had a slower time course to a new equilibrium in both the ascending and descending arms of the curves with significant curve differences (*p* < 0.001 MANOVA) (49). After reaching the endpoint, oscillations were present and were more pronounced in the 30–0 than in the 0–30 mm Hg curves.

Relative velocity was calculated as (ΔSCA/SCAmax)/ΔT. Mean maximum velocity for NL and GL was 0.031 ± 0.002 and 0.024 ± 0.006, respectively, in normalized procedure-defined units (p.d.u). The velocity differences did not reach significance (*p* < 0.304) (Supplementary Figure 2).

## Discussion

Volumetric 3D HR-OCT images permit us to characterize 3D architecture, relationships, and surface topology while concurrently imaging structural features inside the same pathways. Our study demonstrates that pulse-dependent changes in lumen dimensions can be quantified and are synchronous. We find reduced motion responses in GL eyes.

### Surface Topology and Internal Structure of Outflow Lumen Pathways

#### Septa, Collector Channels, and Circumferential Vascular Channels: Schlemm’s Canal Outlet Valves?

**FIGURE 9:**
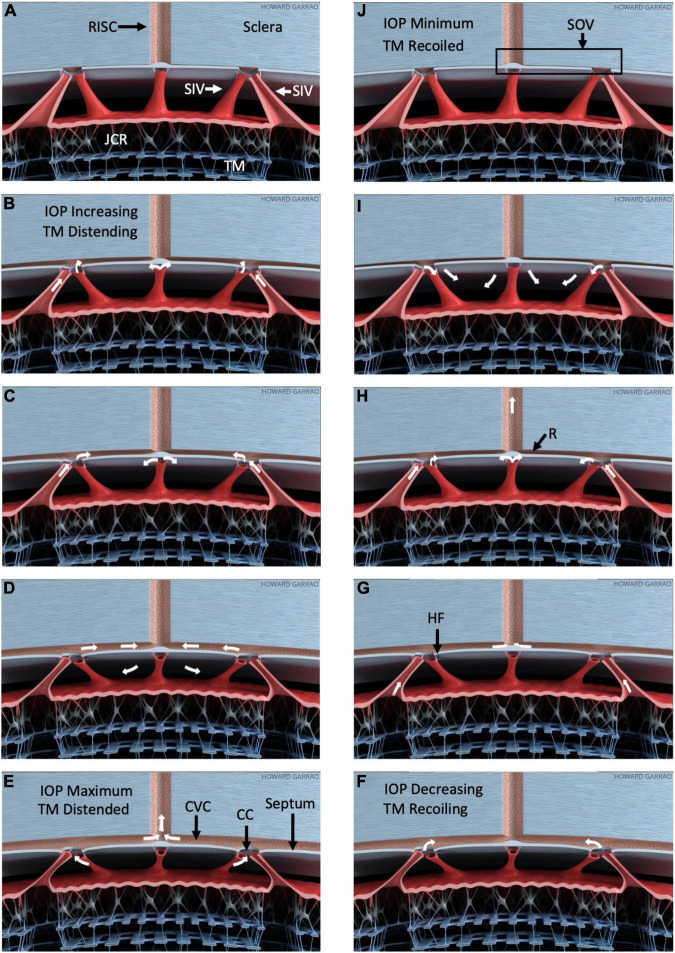
Pressure-dependent outflow system tissue motion and flow. Schematic depiction of outflow structure pressure-dependent motion and related outflow oscillating around a mean setpoint (Supplementary Video 4). **(A–E)** Depict trabecular meshwork (TM) distention into SC in response to a pressure increase. **(F–J)** Depict recoil from the distended configuration. As IOP increases **(B)**, the TM and juxtacanalicular region (JCR) progressively enlarge, decreasing SC dimensions. SIV connect the TM to septa that appear as hinged flaps (HF), where they connect to CC entrances. The SIV is obliquely oriented within the circumference of SC. Outward TM motion can cause the SIV to induce tension on the septa and hinged flaps at collector channels (CC). Thin mobile septa provide a barrier between SC and circumferential vascular channels (CVC) adjacent to the canal. As pressure falls, the CVC progressively closes with a resulting increase in resistance (R), as noted in **(H)**. The resulting behavior permits the CVC region to act like an SC outlet valve-like structure (SOV). Progressive closure reduces inward flow from SC and outward flow to radially oriented intrascleral vessels (RISC). IOP-dependent TM movement can act as a sensor controlling the SOV configuration, thereby providing a feedback loop to control IOP. Minimal linear TM motion may amplify flow resistance because of Poiseille’s law. White arrows depict one possible aqueous flow sequence. All aspects of the model are provisional and subject to modification.

Figure 9 provides a schematic view of structural relationships, motion, and possible aqueous flow patterns. Recent technological advances have resulted in continual modification of the depicted model, which is provisional and expected to require continued changes. We found evidence that the CCs leave SC and pass a short distance through the scleral collagen of septa. The CC then joins circumferentially-oriented vascular channels or CVC closely adjacent and parallel to SC. Figures 2–4 emphasize the orthogonal orientation of the exit of CC from SC and their entry into the CVC lumen. Prior reports have referred to the CVC as a circumferential deep scleral plexus using the acronym CDSP (7) or deep scleral plexus (DSP) (33). As the role of these newly recognized structures comes into more explicit focus, it would be valuable to adopt a term that reflects their physiologic significance as a secondary compressible chamber that can modulate aqueous flow.

Figures 2, 4 demonstrate how the lumen of the CVC provides communications between adjacent CC. Scleral attachments suspend thin, mobile septa between SC and CVC. Openings in these mobile septa, which we call collector channels, provide aqueous access to the CVC lumen. When viewed in cross-section, regions of septa surrounding CC form what appear to be hinged collagen flaps at CC entrances; evidence to support these findings is from the dissecting microscope (5, 32), light microscopy (20, 32, 50, 51), and confocal fluorescence (7). Further support for the distal pathway findings is evidence from scanning electron microscopy (5–7, 21, 32, 33, 44, 52), microvascular casting involving through-cornea imaging (7, 33), and HR- OCT studies (21, 43, 44). Additionally, microCT studies document pressure-dependent CC configuration changes and abnormalities in glaucoma (9, 53).

As documented in the above references, the septa mobility permits their pressure-dependent movement into the CVC lumen that can act as a second dynamically compressible chamber. When the septa compress this secondary chamber, aqueous cannot pass into the CVC from CC or exit into radially oriented, more distal channels. Evidence of the septa-CC-CVC constellation of anatomic features and related motion has led to the proposal that the arrangement may have the ability to act as an SC outlet valve (7). The SOV term encompasses the anatomic features of the CC, septa, and CVC. Their related motion permits the SOV structural elements to act in concert opening and closing CC entrances and the CVC lumen. Figure 9 illustrates the motion permitting the structural features to function as Schlemm’s canal outlet valve-like structures.

The CVC-dependent communication between adjacent CC may explain our finding of a distended CC directly beneath a cannula area that occludes the CC lumen. Despite the cannula blocking the CC exit site from SC, aqueous can reach a CC distal to the cannula and then, through the communicating CVC, fill the CC beneath the cannula (Figures 2, 4).

#### Schlemm’s Canal Inlet Valve-Like Structure Properties and Relationships

Comparison of surface topography with sections through regions of the volumes oriented in multiple planes (Figure 5) permits an improved understanding of SC, SIV, CC, and CVC relationships. We find the SIV connecting the TM and CC originate as a funnel-shaped extension of SC inner wall endothelium. The SIV cross SC to join hinged flaps at SC external wall.

Previous studies have shown that the SIV endothelial wall is continuous with SC inner wall endothelial cells and develops a funnel-shaped protrusion into SC. The SIV then narrows to form an endothelial-lined lumen that crosses to SC external wall as shown by light (5, 24), transmission (6), and scanning electron microscopy (5, 6).

Increasing pressure in the canal causes its lumen enlargement. The SIVs experience increased stress as their attachments to the TM and SC external walls become progressively separated. The increasing tensile stress causes the cylindrical portion of the SIV to experience increased strain manifest by their axial elongation and radial narrowing (6, 21, 24).

Schlemm’s canal-CC height and SIV elongation have been compared using real-time, same sample OCT imaging during pulsatile aqueous infusion into SC. The motion of SC, CC, and SIV was synchronous. Each of the structures had a rapid increase in height over the 1st 150 ms, at which time the SIV elongation reached a plateau. The SC and CC height slowly increased over a 400-ms interval (21).

Immunohistochemistry confirms structural features, relationships, and wall composition like the SC inner wall (54). The SIV and juxtacanalicular region lumen are continuous and contain comparable cells, demonstrated with light and transmission electron microscopy (6). The loosely arrayed, amorphous extracellular matrix material (ECM) in the SIV lumen differs from the ECM of dense collagenous septa arising from the SC external wall. Serial sections regularly reveal a cylindrical appearance like a tube (6, 24, 55). Pressure-dependent dimension changes make the presence of a lumen clear (Figure 5).

Current OCT technologies do not have a combination of magnification and penetration necessary to image SIV or SOV dimensions from the scleral surface. We circumvent the constraint by imaging from the TM surface while controlling SC pressure gradients. The protocol results in SC dilation that straightens the SIV and stretches them directly across SC. The platform allows us to identify tissue connections, stresses, and strains associated with boundary conditions when SC pressure is higher than AC pressure.

Schlemm’s canal pressurization provides information about relationships but does not simulate *in vivo* positive pressure IOP that causes the TM to move outward into SC. The SIV is oriented circumferentially in the canal at positive pressures. Increasing tension will be placed on the obliquely-oriented SIV as the TM moves outward into SC. The resulting tension increase will result in stresses that induce SIV strain, causing them to elongate. The SIV elongation will result in strain manifest as deformation of the flap-like entrances of CC ostia. In this proposed model, the increased systolic stress thus pulls the CC entrance flaps open during systole. The behavior is illustrated in Figure 9.

### Synchronous Trabecular Meshwork and Distal Tissue Pulsatile Movement Reveal a Unified Organ System

Laboratory and clinical evidence permit the proposal that aqueous outflow regulation is not determined by resistance control limited to a single location. Instead, tightly linked components of the entire outflow system change shape in unison in response to pressure changes. The lumen dimensions of the tissues along the pathway enlarge as IOP increases and decrease as the tissues recoil. The innate behavior provides a feedback loop increasing aqueous flow as IOP rises while reducing it as IOP falls (6, 7, 32, 33).

Although acting in concert, each component has sensory and motor features that add to the organ system’s ability to achieve exquisite control of IOP. The TM response to motion provides a baroreceptor-like sensory role while at the same time subserving a motor function with its shape change (20); TM distention enlarges the intertrabecular spaces, enlarges the funnel entrances of the SIV (6, 24), and can increase tension on the hinged flaps at CC entrances (21).

As IOP increases, the increased stress results in increased strain, causing the cytoskeleton of the cells to experience increased strain deformation providing a sensory stimulus (7). The propagating aqueous wave passing through the SIV provides shear stress. As the pulsatile wave streams into the canal, it provides shear stress to the SC endothelium (6). Changes in SIV wall shape provide a motor function by increasing the passageway dimensions for aqueous flow (6, 24). SIV also has classic features of Starling resistors that provide a motor function in controlling flow throughout the vascular system (56).

The hinged flaps at CC move in response to IOP changes providing a sensory stimulus (21, 43). Simultaneously, changes in dimensions provide a motor role able to control aqueous flow. Pulse-induced alterations in flow through the CC and CVC provide sensory shear stress (6) recognized as able to provide motor control by NO-induced changes in lumen dimensions (6). The clinical observation was confirmed in cultured endothelial cells (57), porcine organ cultures (58) and in mice (59).

Our MIGS-like platform used HR-OCT studies of steady-state and dynamic motion to identify four types of rapidly moving pressure-dependent outflow structures in humans, (1) the TM that changes SC lumen area, (2) hinged collagen flaps at CC entrances that change CC lumen area, (3) Thin septa that change the dimensions of circumferentially-oriented intrascleral channels adjacent to SC, and (4) SIV suspended between the TM and the hinged CC flaps; SIV exhibit both axial elongation and lumen dimension changes.

The SIV lumen walls’ endothelial cells have elastance properties like the SC inner wall. The SC inner wall endothelial cells have a robust cytoskeleton, well-illustrated by their ability to distend to form large pseudovacuoles (giant vacuoles) involving the entire cell in response to an IOP increase documented by *in vivo* studies. Reduction of IOP results in recoil, with the cells becoming round with the complete elimination of the distended appearance. The behavior demonstrates the elastance properties of the cells, the ability to rapidly distend and recoil, and accruing and releasing elastic energy.

The SIV exhibit similar elastic behavior, exhibiting deformation in both their radial and axial lumen dimensions. As IOP increases, steady-state SC lumen dimensions increase, requiring alterations in dimensions of the SIV (21). When experimental conditions cause a rapid increase or decrease in SC dimension, axial elongation and shortening of SIV occur within milliseconds, as demonstrated with high-resolution OCT (21). Videography during operating room microscopy provides direct *in vivo* observation of the highly elastic structures that undergo marked elongation and recoil. SIV structural failure occurs *in vivo* only after marked axial stretching. Aqueous gushes from the disrupted ends (6).

By demonstrating a TM-hinged flap linkage, we identify the SIV as structures capable of synchronizing TM and valve-like hinged flap motion. Disruption of the SIV connections to CC entrances reduces the ability of CC to respond to pressure changes. The altered CC responses were demonstrated using a Harvard perfusion pump to experimentally determine the volume and speed of fluid introduction sufficient to overexpand SC with associated disruption of SIV (46).

Direct observation provided by high-resolution OCT provided real-time observation of SIV elongation, thinning of the central portion of the connection, followed by complete disruption and retraction of the disrupted SIV ends. During SIV disruption, the HCF, no longer under tension, fell back against SC external wall. Before SIV disruption, increasing steady-state SC pressure demonstrated that the HCF moved into SC in response to SC expansion. After SIV disruption, no expansion-dependent HCF movement was present (46).

The TM’s pressure-dependent tensile loading provides continuous prestress permitting responses to mean and oscillatory IOP changes. SIV is oriented obliquely in SC circumference at physiologic pressures. Physiologic TM distention can establish tensile stress on the SIV and their attachments to hinged flaps at CC. TM distention and recoil can thereby determine CC entrance dimensions, an intrinsic long-recognized relationship (7, 50).

Non-human primate outflow studies identified similar structures, rapidity of motion, and associated changes in lumen size (21, 60) that our current study identified in human tissue. The constellation of synchronous structural movement caused a rapid change in pathway lumen size. Structural motion and lumen path dimension changes can explain and predict the clinical findings of the pulsatile flow of aqueous into the episcleral veins in humans (41). Identifying the motion of the TM and CC entrances in humans provides new opportunities to assess motion differences between NL and GL eyes and MIGS impact on the movement.

### Schlemm’s Canal and Collector Channels–Synchrony of Wall and Lumen Dimension Changes

The real-time, same sample imaging of our study quantitates the changes in both SC and CC lumen dimensions simultaneously. Synchrony of SC and CC lumen dimension changes results from the concurrent motion of the TM and the septa that surround the lumen of CC entrances.

Human *in vivo* studies indicate that the SC lumen behaves as a compressible chamber (17, 42). Chamber dimension changes result from pressure-induced TM motion. IOP-induced stress results in TM strain; the lamellae and SCE experience progressive deformation as IOP increases within the physiologic range (20, 26). Physiologic IOP is a loading force resulting in continuous TM prestress. The elastance/stiffness properties of the TM counterbalance the IOP-induced load. The balanced loads result in equilibrium; the TM remains poised between the anterior chamber and SC at its optimized mean IOP setpoint. The pressure-induced prestress permits the TM, SIV, and hinged flaps to respond rapidly to changes in IOP.

Recent evidence suggests that the mobility of the septa between SC and the CVC lumen permits the CVC to act as a second pressure-dependent compressible chamber. The hinged flaps at CC represent portions of the septum walls of CVC that surround CC entrances. Septa outward movement closes both CC entrances and the CVC lumen. In this conceptual framework, the prestressed tensionally integrated relationship of the TM, SIV, and hinged flaps at CC entrances permit synchronous TM and septum/hinged flap motion. Septum motion can thereby act as an SC outlet valve, governed by pressure-dependent TM distention. The arrangement can act as a fluidic control system with on-off, fluidic amplifier, and fluidic logic gate properties.

Both steady-state and real-time imaging identify TM-SIV-HF tissue linkages, including progressive elongation of SIV and an increase in their lumen size as transtrabecular pressures increase (Figure 5). The endothelial lined tubes of the SIV elongate and shorten at the same speed as SC and CC lumen dimension changes. The changes in SIV axial length can assess the stiffness of SC endothelial cells in isolation without the confounding cellular, and ECM connections to the juxtacanalicular region found at the SC inner wall.

### Distention and Recoil Speeds Sufficient to Respond to Pulse Transients

Outflow system fixation at steady-state pressures can identify the ability of the tissues to deform, but the rapidity of responses cannot be determined. We use a recently developed platform using an aqueous solution to change transtrabecular pressure gradients. Simultaneous monitoring with HR-OCT during pressure changes now permits addressing the question of response rapidity.

An increase in lumen dimensions of SC from a relatively collapsed to a dilated configuration occurs within ≲200 ms when we induce an increase in SC pressure. After enlargement, lumen size reduction occurs at similar speeds. The changes in steady-state dimensions with distention and recoil were each highly significant. The SCA increase results from the external experimentally-induced force of IOP deforming the trabecular lamellae. The distension and recoil responses represent an intrinsic trabecular lamella biomechanical property that stores and releases elastic energy.

*In vivo*, the mean oscillatory behavior in response to the ocular pulse is 60 beats/min, providing a cyclic duration of ∼1,000 ms. The rapidity of change in the SC and CC area indicates that the biomechanical properties of the tissues permit them to respond to oscillatory changes efficiently within the pulse-dependent time frame.

### Motion Duration—Synchrony of Schlemm’s Canal-Collector Channels Motion

A comparison of the duration of the TM and CC tissue motion responses revealed that full distention occurred in both tissues within about 138 ms and was not significantly different. Recoil occurred in the TM within 178 ms and the CC within 186 ms, again not significantly different. The duration of motion of the ascending and descending TM curves were not significantly different, but the CC curve differences approached significance (*p* = 0.06). The comparable onset and duration of SC and CC dimension changes provide quantitative evidence consistent with the synchronous behavior observable in real-time videos (Supplementary Videos 1–3 and Supplementary Table 3).

### Bulk Trabecular Meshwork Motion—A Tissue Subjected to 30 Million Cycles per Year

The resolution of HR-OCT imaging is sufficient to measure the bulk motion of the trabecular beams but is not adequate to resolve SC endothelial motion. The similarity of the ascending and descending motion curves suggests that the intrinsic bulk motion of the TM lamellae tightly links their elastance/stiffness properties. The TM beams are composed primarily of collagen and elastin, which determine their biomechanical properties. Investigators liken the TM beam composition to a tendon that regularly undergoes distention and recoil (61). The TM beams experience a continuous mean pressure load and oscillatory stresses that occur ∼60 times per minute, like the papillary tendons and aorta. About 30 million such oscillatory events happen in a year.

### Pulse Transients and Continually Recurring Physiologic Schlemm’s Canal Closure

Blinking and eye motion cause transient IOP elevations from the mean baseline pressure of 16 mm Hg 10 mm, resulting in about 25 mm Hg IOP transients occurring multiple times per minute while awake. *In vivo* studies show pressures of 25 mm Hg cause the TM to distend into SC, narrowing its lumen. As pressure increases further, the lumen becomes occluded, resulting in extensive apposition of SC walls (20, 26). Our study confirms prior reports demonstrating that the TM can undergo full excursions within 100–200 ms.

The combined evidence suggests that the TM continually experiences large transient deformations that, during waking hours, can briefly occlude much of the SC lumen. To maintain an appropriate relationship between SC walls, the TM must then be capable of rapidly recoiling from its distended configuration. The biomechanical properties permitting rapid distention and recoil require the storage and release of elastic energy. Elastance/stiffness curves (43, 46) and inverse finite element modeling (45) may be used to characterize the human outflow system’s bulk TM motion properties.

### Tissue Motion and Aqueous Flow During Systole and Diastole

The cardiac-dependent systolic and diastolic relationship to pulsatile aqueous flow is well established. Moreover, recent studies document pulse-dependent TM motion. Pulsatile aqueous waves seamlessly propagate through the outflow system during the cardiac cycle. However, a definitive characterization of the aqueous-flow-tissue-motion sequence awaits improved technology. Figure 9 and the animation in Supplementary Video 4 depict one possible relationship.

Laboratory evidence to develop the proposed animation sequence is based on experimentally induced pulsatile TM, SIV, and SOV motion visible at the dissecting microscope. Further evidence involves high-resolution OCT imaging of outflow system motion. The motion is induced by creating transtrabecular oscillations or pulse transients from both the AC and SC.

In the proposed aqueous-flow-tissue-motion sequence, AC pulse pressure increases during systole, and the TM moves outward, resulting in a corresponding increase in SC pressure. Fluid initially moves through the SIV, but SC pressure surrounding the SIV limits its lumen size, preventing further aqueous entry into the canal as pressure rises. As the systolic pulse wave moves the TM outward into the canal, obliquely-oriented SIV elongates resulting in increased tension that pulls the SOV open. The SC compression by the distending TM results in increased pressure forcing fluid out of the canal through the open SOV.

When the TM recoils during diastole, pressure in SC decreases. The pressure surrounding the SIV in SC also decreases, leading to increased aqueous flow into the SIV; simultaneously, the falling SC pressure favors aqueous flow from the SIV into SC during TM recoil. The recoiling TM also reduces tension associated with the systolic induced circumferential elongation of the SIV. Reduced SIV tension in diastole results in SOV closure until the next systolic cycle begins.

### Trabecular Meshwork Motion Differences—Normal and Glaucomatous Eyes—Elastance/Stiffness Considerations

When comparing the 0 baseline and 30 mm Hg SC area steady-state configurations, the differences were significant in both the NL and GL segments (*p* < 0.0001). However, baseline SC area was much smaller in NL than GL eyes (mean SCA 1,294 vs. 6,502 μm^2^, respectively), while maximum SCA was similar (mean 30 mm Hg SCA 13,343 vs. 15,025 μm^2)^. Total SC area changes were more significant in the NL than in GL eyes (12,549 vs. 8,523μm^2^) (*p* < 0.0001).

One explanation for the disparity between the initial SC dimensions and excursions may relate to differences in elastance/stiffness. These *ex vivo* segments no longer have a ciliary body attachment. Without ciliary body tension, the intrinsic ability of the normal TM to recoil often results in almost complete closure of SC, a finding in many of our segments. If tissue is stiffer in GL eyes, one would anticipate a reduced ability to recoil, thus resulting in a larger canal at baseline. Similarly, our study also finds that increased GL stiffness may reduce the TM’s ability to undergo excursions in response to SC pressure changes.

### Minimally Invasive Glaucoma Surgery: Relevance of Outflow System Biomechanics to Clinical Medicine

Minimally invasive glaucoma surgeries can impact the distal outflow system in several ways. At the site of a MIGS stent insertion, our study demonstrated that the device compresses the TM and SC external wall as illustrated in Figure 2, consistent with previous reports (5, 21, 62). However, immediately distal to the ≈200 μm diameter MIGS-like device entry site region, the SC lumen is held open. Holding SC open beyond the device end can improve aqueous flow to distal CC entrances (Figure 2 and Supplementary Videos 1–3). Pulse waves from the MIGs-like device propagate along SC circumference, causing rapid distention of the canal walls, tension on SIV attachments, and enlargement of CC entrances. Our study showed that a pulse wave originating from the MIGS-like device is capable of opening a CC and more distal radial intrascleral outflow channel beyond the location of an intervening SC constriction (Supplementary Video 1).

Laboratory studies have long recognized that under steady-state conditions, CC entrances can be held open by attachments between the TM and hinged flaps at CC entrances (50). *In vivo* non-human primate studies demonstrate that increasing IOP causes TM distention that occludes SC (20, 63), and TM tissues herniate into CC entrances (23), a finding later confirmed to be similar in *ex vivo* bovine eyes (64). *In vivo* pressure gradient reversal eliminates SC closure and herniations (20, 63). Our study shows that an SC MIGS device that communicates with SC can result in CC opening and closing rapidly within the time frame of the ocular pulse and ocular transients. Modulation of TM lamellae elastance determines the TM relationship with SC external wall. The outflow abnormality in glaucoma may result from altered TM elastance/stiffness properties. The altered elastance/stiffness can result in loss of the TM’s ability to maintain (1) optimal mean SC chamber dimensions and (2) responses to ocular transients. In our study, the insertion of a MIGS-like device bypassed the TM, providing a means for pulse waves to directly enter and dilate SC, followed by opening more distal CC entrances.

## Limitations

The small sample size is a limitation of this study, so the clinical significance of the findings must be interpreted with caution. Correlation with alternative imaging methods such as scanning electron microscopy, immunohistochemistry, confocal microscopy, and tracer studies would assist in validating our work. Such studies are beyond the scope of this report. However, these modalities are the subject of prior publications, each of which provides unique images documenting structural relationships (5–7, 20, 24, 32, 33).

It may be argued that cannulas used in this study do not simulate a MIGS-like device. Our cannula was fashioned from PE60 tubing with a 1220 μm ID tapered to ∼200 μm where it entered SC and further tapered to ∼150 μm over a 350 μm distance (Figure 3). Resistance varies with the fourth power of the radius, so the most relevant resistance will be in the 350 μm region that tapers from 200 to 150 μm ID. The above dimensions suggest that our MIGS-like device reasonably approximates the dimensions of commercially available widely used stents.

Such stents include (1) The Istent with an L-shaped configuration. A 250 μm snorkel passes through the TM to SC with a 330 μm outside diameter (OD) and an inside diameter (ID) of 120 μm. The snorkel joins a 1 mm long body in the canal with an open half-pipe configuration facing SC external wall, (2) The single piece iStent Inject has a 360 μm length, a flange residing in the anterior chamber, a thorax resting in the TM, and a head residing in SC. The head and flange each have a maximum OD of 230 μm OD. The 360 μm long central conduit has an 80 μm ID and communicates with four 50 μm ID side flow outlets, (3) The Hydrus Microstent is 8 mm long with an open scaffold design having a major axis of 295 μm and a minor axis of 185 μm with the open surface facing SC external wall.

Possible confounding issues in comparing NL and GL eyes include differences in age, time from enucleation to initiation of organ culture, and the time to initiation of OCT experiments; these variables were not significantly different (Supplementary Table 1). Postmortem eyes experience issues with autolysis and are without a physiologic milieu until placed in organ culture. The postmortem status warrants questioning whether the tissues will have physiologic responses to pressure changes. The media we used restores or recovers tissues to maintain signaling parameters similar to those present *in vivo* (65). The human eye response rapidity is similar to freshly enucleated primate eyes (16). Nonetheless, possible alterations in the biomechanical responses warrant further exploration.

Our study protocol reverses pressure gradients and is unlike what investigators customarily simulate in outflow studies. The approach is novel and adds new information not obtainable by other means. Nevertheless, the approach warrants an explanation.

Our test conditions replicate what occurs during physiologic activities such as yoga and gymnastics. Transitioning to an inverted posture results in the blood rapidly filling SC with synchronous changes in pressure gradients before brief achievement of a new inverted steady-state configuration (22). Inversion causes pressure to rise as high or higher than 30 mm Hg, and blood refluxes into SC. For blood to enter the SC, pressure in the canal must be higher than the 30 mm Hg pressure found in the AC (66). This report limits the physiologic pressure reversal to 30 mm Hg which is less than the maximum SC pressures documented in normal activities.

Inversion of thick-walled hollow tubes is a boundary problem explored in the biomechanics literature (67). Such tubes retain intrinsic residual stress representing the stored energy in a body free from external loads. The relative spatial locations of the inner and outer walls are reversed during the testing. If the wall composition is homogenous, inflation will not provide new information. However, reversal adds new information with non-homogenous wall composition.

Trabecular meshwork lamellae composition is similar in all the beams, but the geometry differs, with the beams closer to the anterior chamber much larger and thicker than those near SC inner wall (68). One might expect circumferential residual stress augmentation with SC pressure gradients higher than the AC. Although residual and loading stresses will be reflective of biomechanical properties, these factors should be considered when evaluating the findings of our study.

Zero mm Hg was chosen as the baseline SC pressure resulting in the SC lumen being very narrow or collapsed. It may have been preferable to use a higher pressure as the baseline to establish a reference configuration, for example, the mean difference between AC and ESV pressure. The physiologic pressure differential between the AC and SC remains an unsettled question. The orthodox assumption is that aqueous passes directly through SC inner wall with no alternative conduit for flow. If the assumption were valid, it would limit the resistance possibilities to an unknown but physiologically significant transtrabecular pressure difference.

Accumulating evidence suggests the limiting assumption of transtrabecular pressure gradients warrants reexamination. The two most physiologically relevant studies have explored the relative AC and SC pressures in primates *in vivo*. These *in vivo* studies found the AC-SC pressure differential was small, 1–3 mm Hg or less, concluding that most of the outflow resistance was distal to the TM (69, 70). The minimal pressure difference did not fit the prevailing narrative of the time, and the important evidence was not retained in later literature reviews.

Studies on Schlemm’s canal inlet valve-like structures require challenging the limiting assumption of passive flow directly through SC inner wall. The relatively frequent and large SIV flow conduits allow aqueous to enter SC without being forced through SC’s inner wall directly. Outward movement of the TM during systole will force the incompressible aqueous into SC toward SC external wall when AC and SC pressures are approximately equal. As aqueous flows from the SIV into SC during diastole, aqueous can rapidly fill the canal with the reversal, requiring a minimal AC-SC pressure differential. These considerations are consistent with Sears and Perkins’ work (69, 70).

The absence of a normal episcleral venous pressure and ciliary muscle tension is another limitation of our report and inherent in all *ex vivo* studies. The SC opening at the cut end of the segment opposite the location of cannula entry was not occluded in our study. However, SC filled along its entire length in the steady-state 3D reconstructions in this study was consistent with prior studies in non-human primates (21) and humans (43, 46).

The platform we developed most closely resembles stents that create connections between the anterior chamber and SC and may be less helpful in assessing the effects of procedures that disrupt or remove regions of the TM. After reaching a minimum or maximum, marked oscillations were present in the TM and CC tissue motion curves. The oscillations may be due to the intrinsic behavior of the tissues or reflected waves in the cannula tubing and warrant further study. The results we present should be interpreted cautiously because of the limitations listed above.

Although we posit the IOP control mechanism depicted in Figure 9, several alternative interpretations can be envisioned for the effects of tissue motion that we find. We recognize the essential synergistic role of the juxtacanalicular region. The system, as proposed, does not require pulsatile behavior but, like the lymphatics, can simply act as a conduit depending on pressure gradients (71, 72). The recurring loops of the collector and circumferential vascular channels are suggestive of Tesla valves (73) that require no tissue motion. Regulatory redundancies may incorporate more than one tissue level mechanism to ensure pressure control.

## Conclusion

Our MIGS-like platform was able to control SC pressure. We dilated SC and used HR-OCT to identify surface topography internal and structural features. Our study identified valve-like structures entering and exiting SC. Systematically changing pressure gradients during HR-OCT imaging allowed us to study outflow system motion. We found the entire outflow apparatus acting as a unified organ system with rapid synchronous dimension changes of proximal and distal pathways. The system’s pressure-dependent changes in dimensions provide a means to control IOP. The motion can also explain and predict pulsatile aqueous outflow to the episcleral veins that become abnormal in glaucoma.

We reported the differences in steady-state and dynamic motion curves. The differences were consistent with abnormal elastance/stiffness in tissue from glaucoma patients. Insertion of a MIGS device into SC bypassed the TM. Direct pulse wave access to SC dilated the more distal lumen of SC and CC. The finding is consistent with the ability of the ocular pulse wave and transients to travel along the circumference of SC to a CC after placement of a MIGS-like shunt.

## Data Availability Statement

The original contributions presented in the study are included in the article/Supplementary Material, further inquiries can be directed to the corresponding author/s.

## Ethics Statement

All tissues were deidentified and complied with IRB requirements.

## Author Contributions

MJ, RW, CX, and EM: conception and design. TA and JV: tissue acquisition, organ culture and database organization. MJ, RW, and CX: experiments, data acquisition, and interpretation. MJ: first draft. JW, RW, and EM: critical revisions. All authors read and approved the final manuscript.

## Conflict of Interest

The authors declare that the research was conducted in the absence of any commercial or financial relationships that could be construed as a potential conflict of interest.

## Publisher’s Note

All claims expressed in this article are solely those of the authors and do not necessarily represent those of their affiliated organizations, or those of the publisher, the editors and the reviewers. Any product that may be evaluated in this article, or claim that may be made by its manufacturer, is not guaranteed or endorsed by the publisher.

## Abbreviations

IOP: Intraocular Pressure
OCT: Optical Coherence Tomography
SD-OCT: Spectral Domain OCT
HR-OCT: High-Resolution OCT
PhS-OCT: Phase OCT
TM: Trabecular Meshwork
SC: Schlemm’s Canal
CC: Collector Channels
SCE SC: Endothelium
SCA SC: Area
NL: Normal
GL: Glaucoma
MIGS: Minimally Invasive Glaucoma Surgery
FEM: Finite Element Modeling
CVC: Circumferentially Oriented Vascular Channel
SIV: Schlemm’s Canal Inlet Valve-like Structures.
