# Supplementary material for: Valve-Like Outflow System Behavior With Motion Slowing in Glaucoma Eyes: Findings Using a Minimally Invasive Glaucoma Surgery–MIGS-Like Platform and Optical Coherence Tomography Imaging
Source: Front Med (Lausanne). 2022 Apr 29;9:815866. doi: 10.3389/fmed.2022.815866 (PMC9099151; doi:10.3389/fmed.2022.815866)

**Supplemental Data**

**Details of Materials and Method**

**Tissue Acquisition and Preparation**

The Oregon VisionGift Eye Bank provided eyes within 72 hours postmortem. All research followed the tenets of the Declaration of Helsinki. The eye bank deidentified records, thereby precluding assessment of details of the glaucoma history. We bisected the eyes and removed the lens, ciliary body, and iris. Anterior segments were mounted on perfusion chambers and maintained for five days in media that provides organ culture conditions demonstrated to restore functional properties in postmortem eyes ^(44)^. Following perfusion, anterior segments were removed and dissected, leaving tissue wedges that retained the limbal region, including cornea and 5-10 mm of the sclera. Segments were placed in culture media and flown on ice overnight to the University of Washington. Wedges of the limbal region with the cornea and 5 mm of the sclera were mounted in a Petri dish with the inner TM surface facing upward (Figure 1A).

A cannula was inserted into the SC lumen and held in place with a micromanipulator. A three-way valve connected the end of the cannula opposite the canal lumen to two media-containing reservoirs that controlled the hydrostatic pressure in the SC cannula. We systematically controlled the SC image location using a micrometer stage attached to the undersurface of the Petrie dish. The high-resolution OCT allowed us to identify and image one to four locations from each wedge.

**Image Acquisition and Assessment of 3-Dimensional (3D) Volumetric Data**

High-resolution OCT (HR-OCT) 3D volume datasets were obtained from the sample while maintaining a 30 mm Hg hydrostatic pressure gradient. The topography of the surface of the SC, CC, and circumferentially oriented intrascleral vascular channels lumen was delineated by defining a region about 4-5 pixels outside the lumen wall in each frame of the stack, assisted by a 3D view software. The segmentation provided a mask that limited the image to the outlined lumen surface areas and their internal structure within each image stack. Projections of 3D surfaces and internal structures of the SC and CC lumens were then developed, permitting surface shape and internal structure comparisons at each frame location.

**Analysis of 2D cross-sectional time-dependent data**

Twenty locations with collector channel motion were initially identified. Without knowledge of the clinical status of the subjects' eyes, a masked observer identified five locations from NL and ten from Geyes that would be satisfactory for study based on clarity of the HR-OCT images. Analysis of lumen motion was done by measuring SC and CC areas at 5 msec intervals between adjacent B-scans. The CC lumen merged with that of circumferentially oriented intrascleral vascular channels where the two lumens joined. The transition could not be delimited in a binary fashion, so the CC and entrances to circumferentially oriented intrascleral vascular channels were analyzed as a single area when images incorporated both regions.

The HR-OCT cross-sectional images were binarized by setting a threshold intensity to the central value of the difference between the surrounding tissue intensity and the lumen intensity. An algorithm automatically obtained five measurements, which were averaged to provide SC and CC area measurements. The first point at which all subsequent measurements were changing in a single direction defined the initiation of motion.

Six timepoint measurements (each time point measurement was repeated five times) at 5-second intervals were averaged to provide a baseline of the lumen area before initiating a pressure change. Values oscillate for some time following a pressure change precluding the use of an averaging approach to identify the final value. The final values for both the rising and following curves were determined by identifying the first time sequence value (p) less than the unidirectional sequence of values preceding it and using the prior point (p-1) as the final value. For analysis of each group, each 5 msec interval for all samples in the group was summed. Calculations then determined a starting point for each curve that would best approximate a curve overlap that minimized the difference between means for individual data bins in 5-second intervals for the group.

# Supplemental Data Figures

Figure S1A Schlemm’s canal and Collector Channel Area - IOP Change from 0-30 mm Hg

Schlemm’s canal (SC) and collector channel (CC) area change when reservoir pressures switch from 0-30 mm Hg. The areas are normalized and expressed in procedure-defined units (p.d.u.).


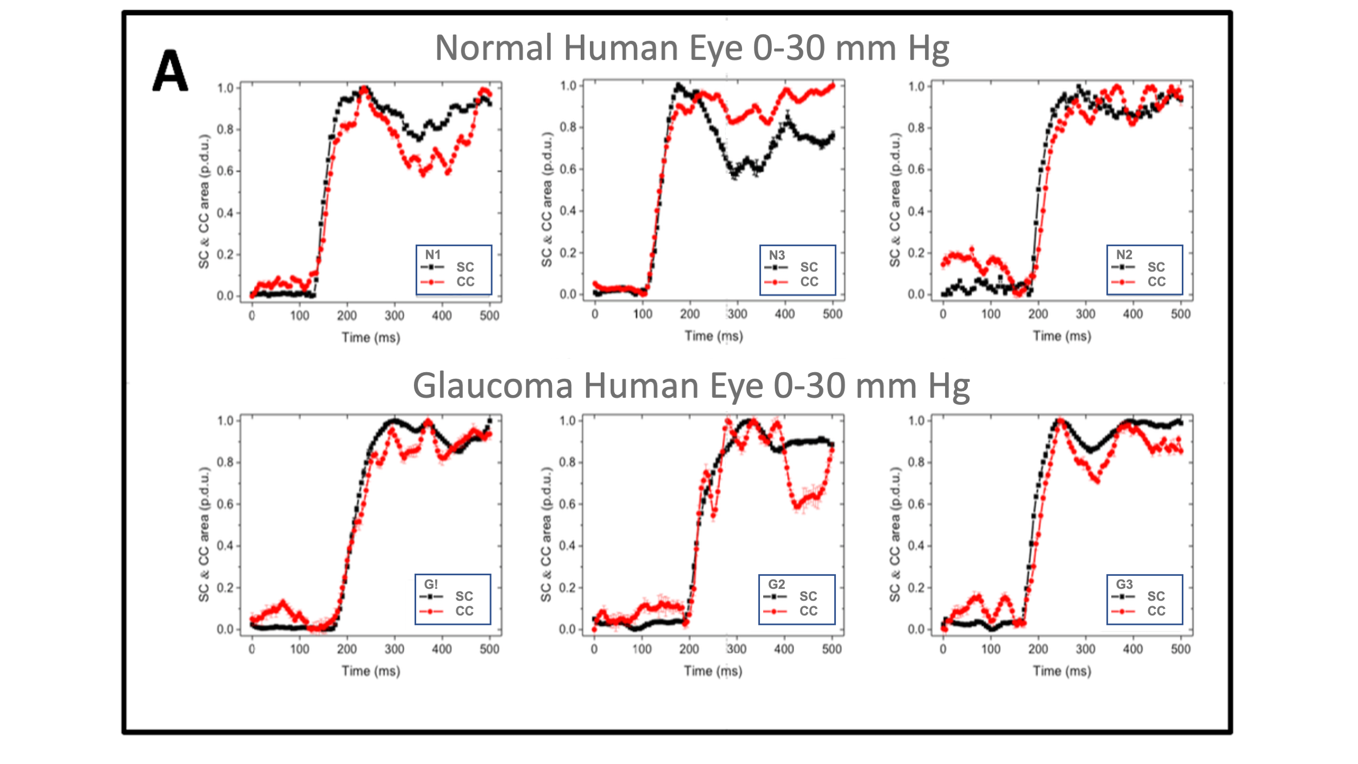

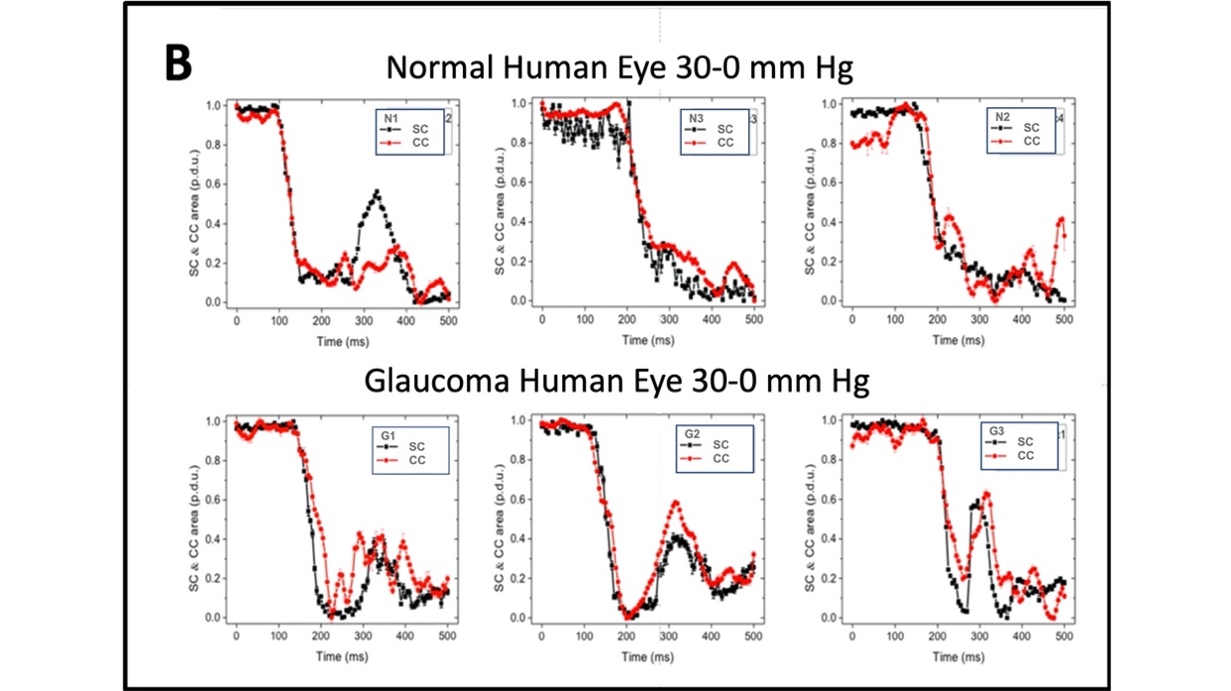


Figure S1B Schlemm’s canal and Collector Channel Area - IOP Change from 30-0 mm Hg

Schlemm’s canal (SC) and collector channel (CC) area change when reservoir pressures switch from 30-0 mm Hg. The areas are normalized and expressed in procedure-defined units (p.d.u.).


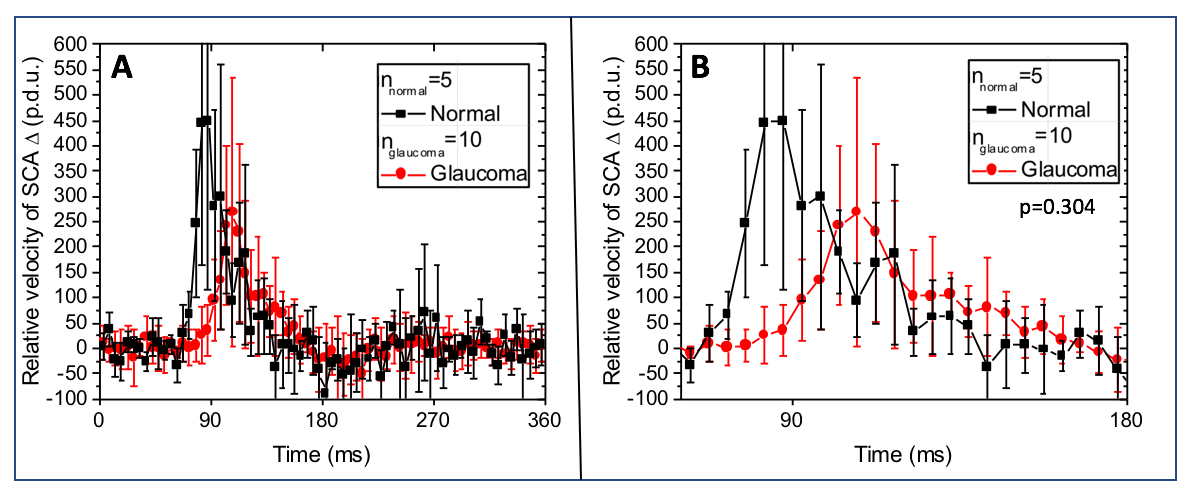


Figure S2 Normalized Mean Maximum Velocity

B is the 60-180 ms segment of the time frame in A.

Table S2 Normal and Glaucoma Schlemm’s Canal and Collector Channel Areas at 0 and 30 mm Hg

Measurements were done at six initial steady-state 5-second intervals at 0 mm Hg and six final steady-state intervals at 30 mm Hg. Each interval was measured five times by an automated algorithm providing a total of 30 measurements for each initial and final SC area analysis.


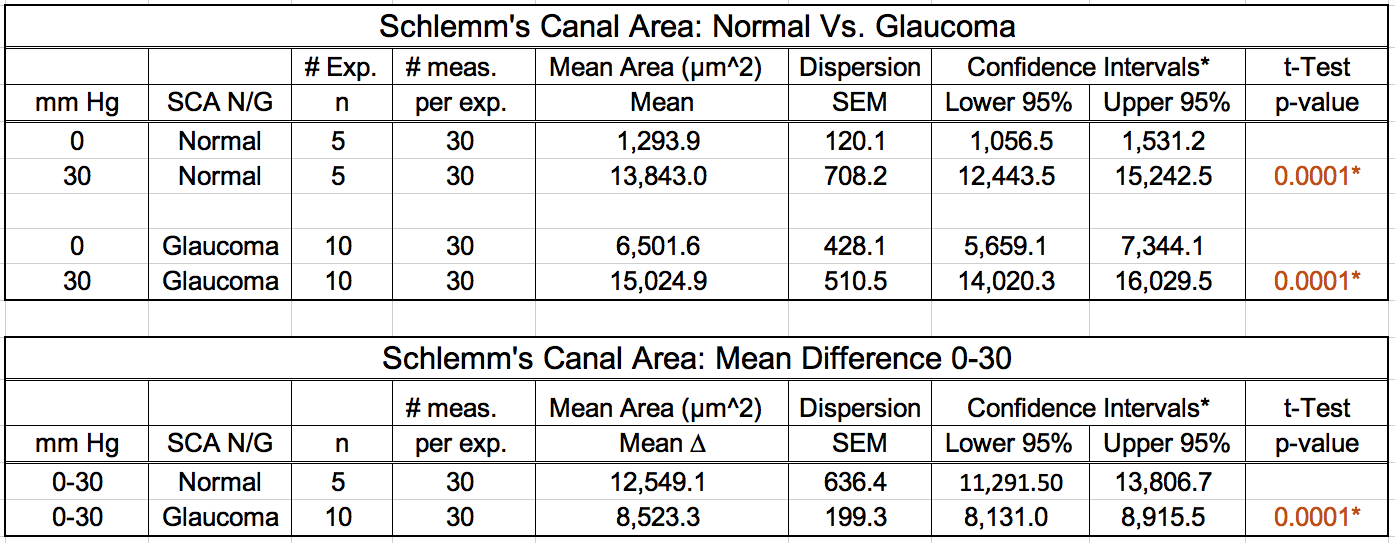


| **Supplemental Data Tables** 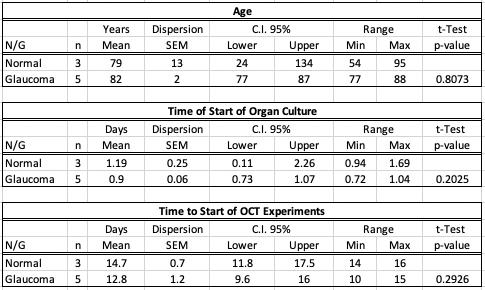 Table S1 Age, Time to Initiation of Organ Culture, and OCT Experiments.  Differences between normal and glaucoma eyes in terms of age, time to start organ culture, and high-resolution OCT experiments were not significantly different. |
| --- |
|  |

## ­

Table S3 Schlemm’s Canal and Collector Channel Pressure-dependent Response Times

Comparisons of Schlemm’s canal area (SCA) and collector channel area (CCA) response times at 0-30 and 30-0 mm Hg for normal and glaucoma eyes revealed a lack of significant differences, as did the pooled normal and glaucoma data. Comparisons of SCA response time differences (∆) at 0-30 mm HG representing pressure-dependent TM deformation and 30-0 representing TM recoil from the distended state were not significantly different. Collector channel deformation times were slower than recoil times and approached significance at p=0.060. All times of distention and recoil were <200 ms, well within the 1000 msec time of the cardiac cycle.


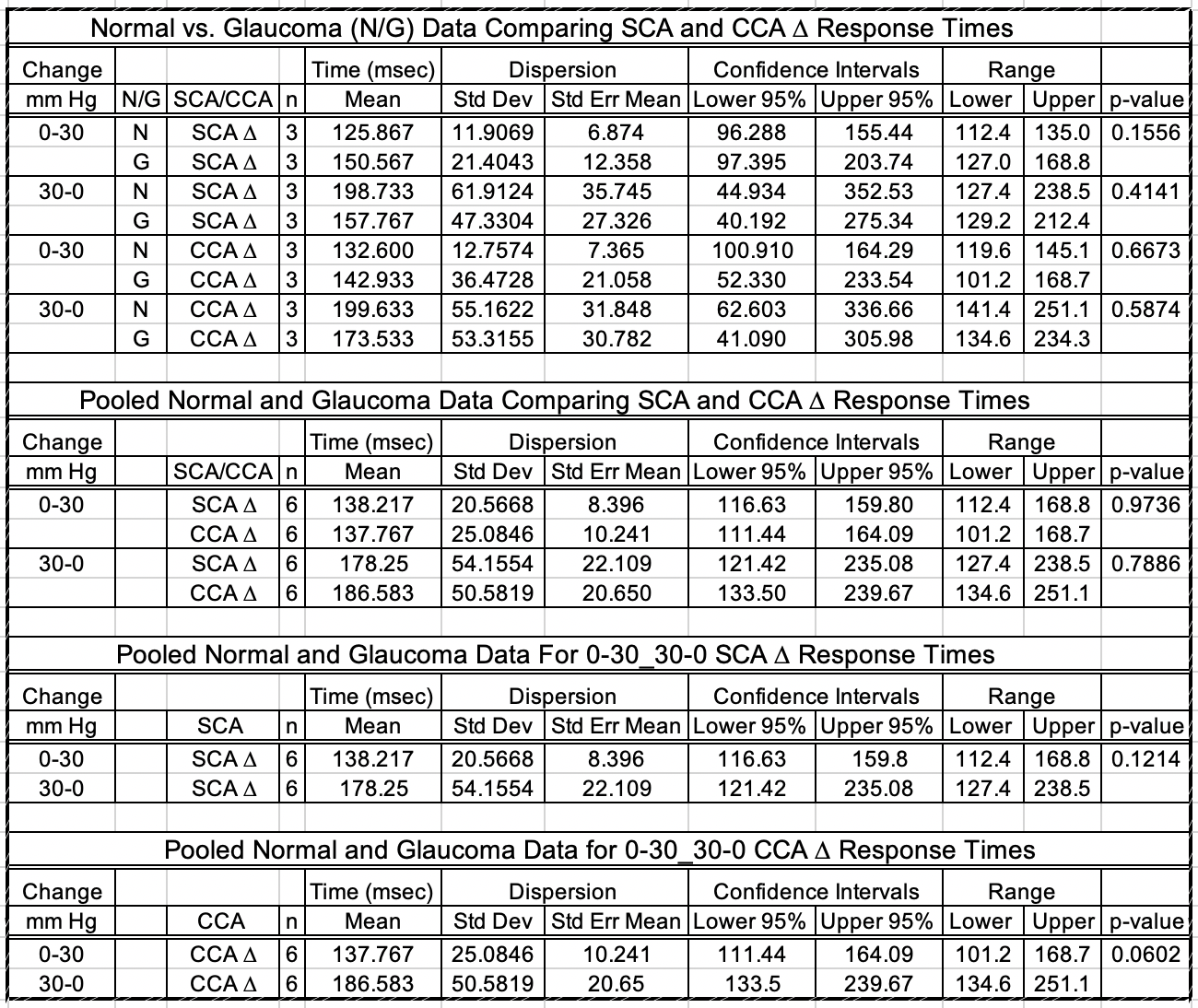

Supplement: Supplementary file 5 [file Data_Sheet_1.docx]
